# Apixaban-Induced Leukocytoclastic Vasculitis: An Uncommon Complication of a Common Anticoagulant

**DOI:** 10.7759/cureus.93407

**Published:** 2025-09-28

**Authors:** Sukhmani Gill, Anushka Verma, Merica Vorachitti, Deepti Boddupalli

**Affiliations:** 1 Internal Medicine, Creighton University School of Medicine, St. Joseph's Hospital and Medical Center, Phoenix, USA; 2 Internal Medicine, Harvard T.H. Chan School of Public Health, Boston, USA

**Keywords:** adverse drug events, adverse effect, apixaban, apixaban-induced leukocytoclastic vasculitis, direct oral anticoagulant (doac), drug-induced vasculitis, leukocytoclastic vasculitis

## Abstract

Apixaban-induced leukocytoclastic vasculitis (LCV) is rare, with only a handful of cases reported. We describe a 68-year-old man who developed a diffuse, painful, pruritic rash and myalgias approximately six weeks after the initiation of apixaban. Extensive infectious and autoimmune workups were negative. Although the skin biopsy was nonspecific, corticosteroid therapy and apixaban discontinuation led to marked clinical improvement. The temporal association and exclusion of other causes support apixaban as the likely trigger. This case contributes to the growing recognition of apixaban-induced LCV and highlights the need to consider drug-induced etiologies in new-onset vasculitic rashes, especially in anticoagulated patients.

## Introduction

Leukocytoclastic vasculitis (LCV) is a small-vessel vasculitis characterized histologically by the neutrophilic infiltration and fibrinoid necrosis of dermal postcapillary venules. Clinically, LCV presents as palpable purpura, most commonly on the lower extremities, and can occasionally be accompanied by systemic symptoms such as arthralgia, abdominal pain, or renal involvement. The etiology of LCV is broad, including but not limited to drugs, infections, autoimmune diseases, and malignancies. About 25% of skin-limited LCV cases are believed to be related to medication, while more than half of the cases have no identifiable cause found [1].

To date, approximately seven cases of apixaban-induced LCV have been reported in the literature. We report what appears to be the eighth such case, contributing to the growing recognition of this rare but potentially serious adverse effect.

## Case presentation

A 68-year-old man with a medical history of Parkinson's disease, hypertension, hyperlipidemia, chronic obstructive pulmonary disease (COPD), and atrial fibrillation presented with a diffuse, painful, pruritic rash, along with worsening shortness of breath. He had undergone successful outpatient electrical cardioversion for persistent atrial fibrillation approximately three weeks prior and had been taking daily 5 mg apixaban for six weeks for anticoagulation.

The patient reported that he noticed the rash approximately two weeks following the cardioversion. It began as a petechial eruption on his arms and legs and progressively spread to involve the trunk, palms, and soles. The rash was associated with diffuse burning myalgias but no fever, chills, joint pain, gastrointestinal symptoms, or recent upper respiratory tract infection. He denied any previous similar rash. There was no history of new medications, recent travel, insect bites, or sick contacts.

**Figure 1:**
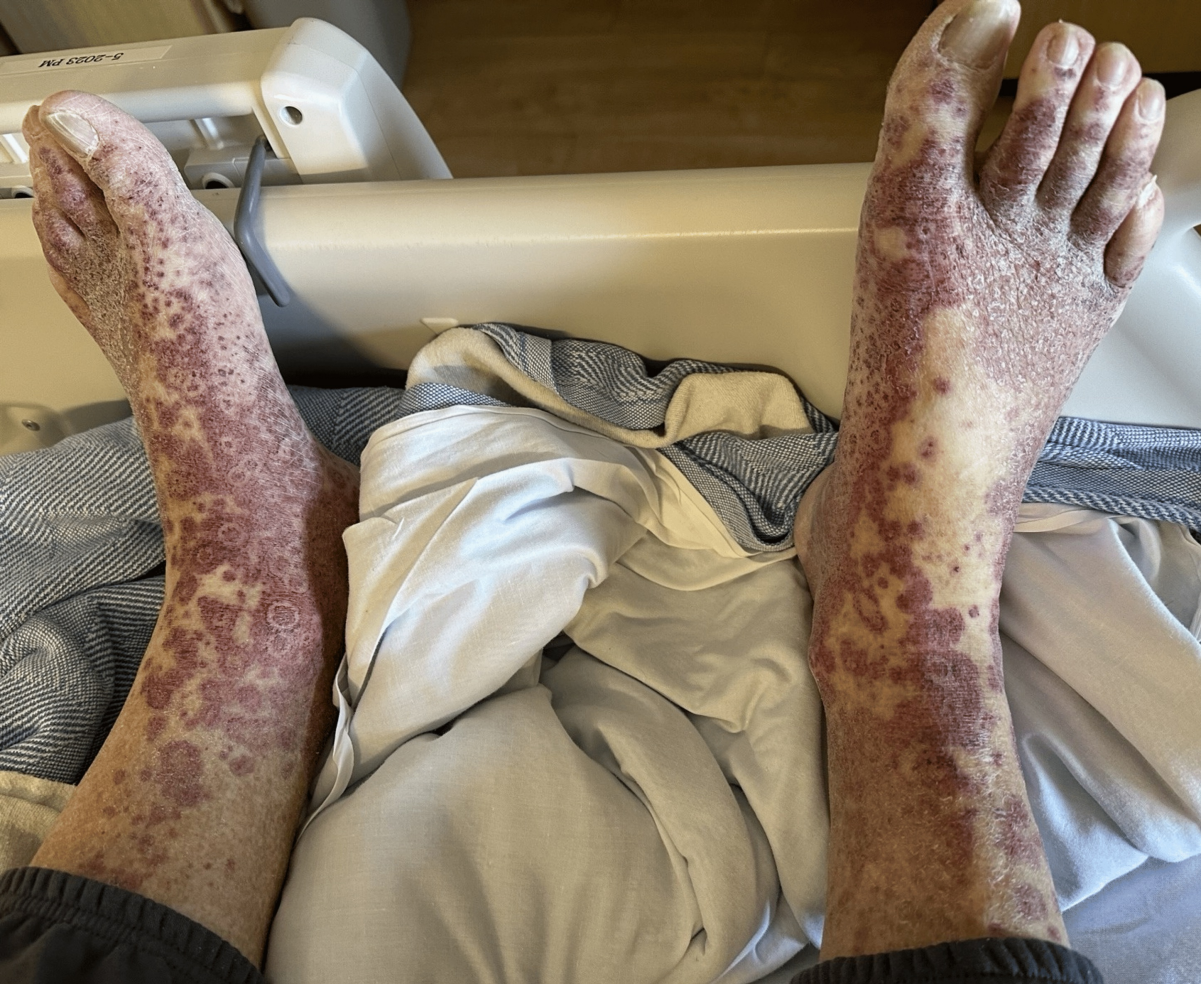
Symmetrical palpable purpuric rash on both legs.

**Figure 2:**
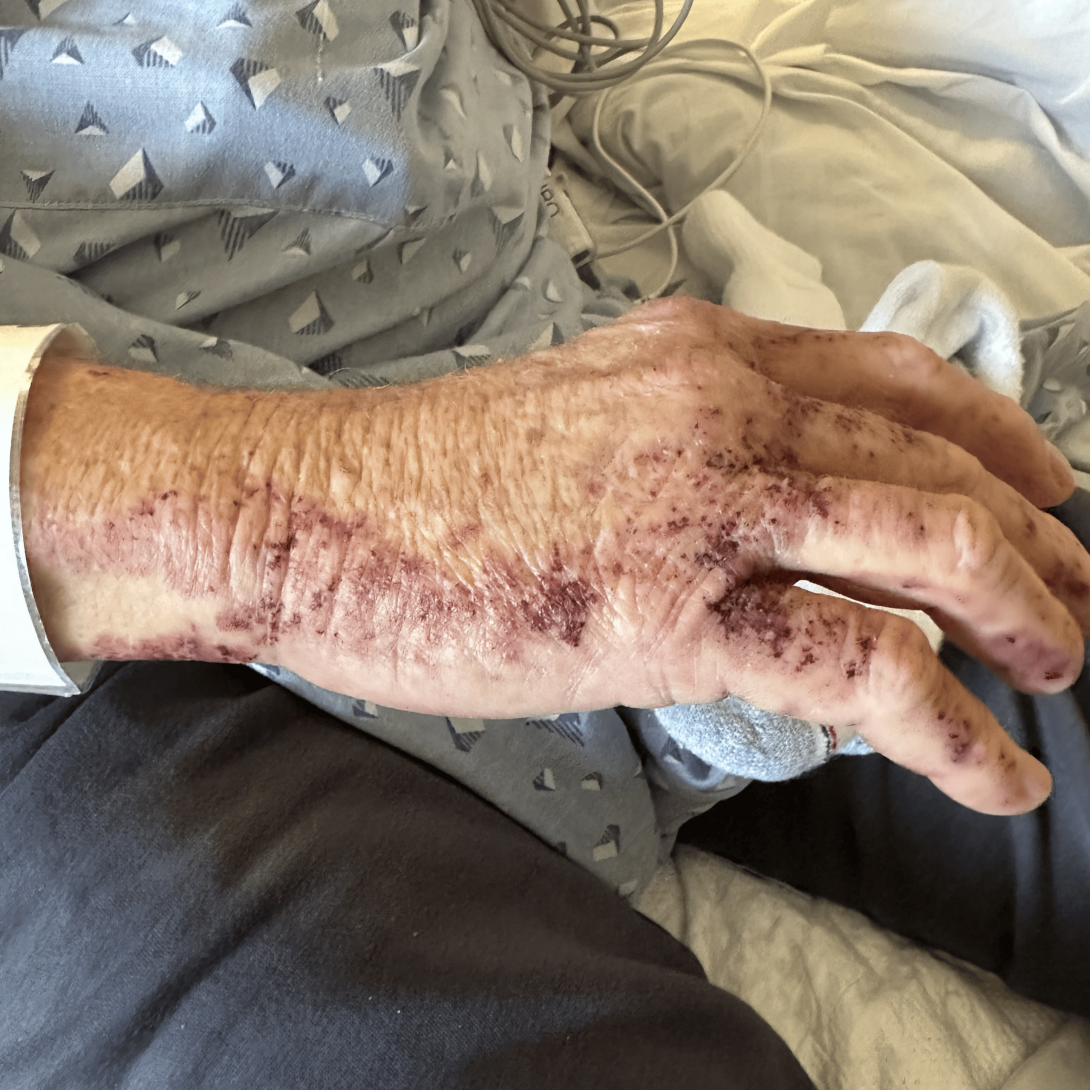
Non-blanching palpable purpuric rash on the right hand.

On physical examination, the patient was hemodynamically stable but tachycardic and tachypneic. Dermatological examination revealed a non-blanching, palpable purpuric rash distributed symmetrically on all four limbs, the trunk, and acral surfaces (Figure 1 and Figure 2). Pulmonary examination was notable for diffuse wheezing consistent with a COPD exacerbation. His home medications included atorvastatin, hydrochlorothiazide, carbidopa-levodopa, gabapentin, and albuterol, all of which had been stable and unchanged for over a year.

Investigations and treatment

Initial laboratory testing showed elevated inflammatory markers, including C-reactive protein (CRP) and erythrocyte sedimentation rate (ESR). A full infectious workup, including human immunodeficiency virus (HIV), hepatitis B and C, herpes simplex virus, parvovirus B19, syphilis, *Borrelia burgdorferi*, Rocky Mountain spotted fever, and coccidioidomycosis, was negative. The viral respiratory panel was also negative. Given the systemic nature of the rash and associated myalgias, an autoimmune workup was pursued.

The patient tested positive for antinuclear antibodies (ANA) and anti-double-stranded DNA (anti-dsDNA) immunoglobulin G (IgG), but complement levels were within normal limits. Myositis and extractable nuclear antigen (ENA) panels, including anti-histidyl-tRNA synthetase antibody (Jo-1), anti-Sjögren's-syndrome-related antigen A (SSA), anti-Sjögren's-syndrome-related antigen B (SSB), anti-topoisomerase I antibody (Scl-70), and anti-ribonucleoprotein antibody (RNP), were negative. Anti-neutrophil cytoplasmic antibodies (ANCA) were also negative. Coxsackie B virus titers were markedly elevated (types 2, 3, 4, and 5), although the patient did not exhibit any clinical signs of viral infection. The patient's key laboratory findings are summarized in Table 1*.*

**Table 1:** Laboratory findings. WBC: white blood cell; ESR: erythrocyte sedimentation rate; CRP: C-reactive protein; BUN: blood urea nitrogen; eGFR: estimated glomerular filtration rate; AST: aspartate aminotransferase; INR: international normalized ratio; PT: prothrombin time; PTT: partial thromboplastin time; ANA: antinuclear antibodies; IgG: immunoglobulin G; dsDNA Ab: double-stranded DNA antibody; IFA titer: indirect immunofluorescence assay titer; Jo-1: anti-histidyl-tRNA synthetase antibody; PCR: polymerase chain reaction; RNP: anti-ribonucleoprotein antibody; RPR: rapid plasma reagin; Scl-70: anti-topoisomerase I antibody; Sm: anti-Smith antibody; SSA: anti-Sjögren’s-syndrome-related antigen A; SSB: anti-Sjögren's-syndrome-related antigen B; ANCA: anti-neutrophil cytoplasmic antibodies; HIV: human immunodeficiency virus; HSV: herpes simplex virus

Test	Patient's value	Reference range
Total WBC count	5.1×10⁹/L	3.6-11.1×10⁹/L
Hemoglobin	13.9 g/dL	12.9-16.1 g/dL
Platelets	200×10⁹/L	150-450×10⁹/L
ESR	112 mm/hr	0-20 mm/hr
CRP	72.7 mg/L	≤10 mg/L
Serum creatinine	2.67 mg/dL	0.65-1.25 mg/dL
BUN	37 mg/dL	8-25 mg/dL
eGFR	25 mL/min/1.73 m²	≥90 mL/min/1.73 m²
Total bilirubin	1.7 mg/dL	0.2-1.2 mg/dL
AST	54 U/L	5-34 U/L
INR	2.0	0.8-1.2
PT	22.6 sec	9.9-13.9 sec
PTT	36.7 sec	25.5-36 sec
ANA (HEp-2, IgG)	Positive, 1:1280 (speckled)	<1:40
dsDNA Ab (IFA)	1:160	<1:10
Extractable nuclear antigen panel (SSA, SSB, Sm, RNP, Jo-1, Scl-70)	Negative	Negative
ANCA (IFA)	Negative, <1:20	<1:20
Complement C3	87 mg/dL	82-193 mg/dL
Complement C4	10.4 mg/dL	15-57 mg/dL
Cryoglobulin	Negative	Negative
HIV 1/2 Ag/Ab	Negative	Negative
Hepatitis B surface antigen	Negative	Negative
Hepatitis C antibody	Negative	Negative
Syphilis (RPR)	Non-reactive	Non-reactive
HSV PCR (blood)	Negative	Negative
Parvovirus B19	Negative	Negative
*Borrelia burgdorferi* Ab	Negative	Negative
Rocky Mountain spotted fever (IgG/IgM)	Negative	Negative
Coccidioides (IgG/IgM)	Negative	Negative
Viral respiratory panel	Negative	Negative
Coxsackie B titers (types 2-5)	1:320 to ≥1:640	<1:10

Given the cutaneous findings and timing, LCV was suspected. A skin biopsy was performed after the initiation of corticosteroids and revealed nonspecific superficial dermal perivascular inflammation without the classical histopathological features of LCV. The biopsy report noted that these findings could represent a resolving drug eruption or viral exanthem and may have been masked by prior corticosteroid therapy.

Hydrochlorothiazide was withheld due to reported hypotension. The patient was initiated on azithromycin for presumed COPD exacerbation and received antihistamines for symptomatic control. Given the strong temporal relationship between the onset of the rash and apixaban initiation, and the lack of other causative agents, apixaban was discontinued. He was transitioned to rivaroxaban for anticoagulation. Systemic corticosteroids were initiated following the exclusion of active infection.

Outcome and follow-up

Following the discontinuation of apixaban and subsequent initiation of corticosteroids, the patient experienced marked improvement in both rash and musculoskeletal symptoms. Because these interventions occurred in close sequence, it is difficult to determine whether resolution was primarily related to apixaban withdrawal, corticosteroid therapy, or both. However, the temporal association with apixaban initiation and the absence of recurrence on rivaroxaban support apixaban as a likely contributing factor. Clinically, pruritus decreased significantly, and his skin lesions began to resolve. His respiratory symptoms improved with supportive care and azithromycin.

The patient tolerated rivaroxaban without recurrence of symptoms. A prolonged corticosteroid taper was prescribed at discharge. The patient was advised to follow up with rheumatology and cardiology and his primary care physician. Based on the clinical course, timing, and exclusion of other etiologies, the findings were consistent with a presumed diagnosis of apixaban-induced LCV.

## Discussion

Apixaban, a direct oral anticoagulant (DOAC) that selectively inhibits factor Xa, is widely used for stroke prevention in nonvalvular atrial fibrillation and for the treatment and prevention of deep vein thrombosis and pulmonary embolism [2]. While bleeding remains the most frequently reported adverse effect, rare hypersensitivity reactions, including LCV, have been increasingly documented [3].

LCV is a histopathologically defined small-vessel vasculitis primarily involving the skin, characterized by neutrophilic infiltration and fibrinoid necrosis. Drug-induced LCV is most often associated with beta-lactams, nonsteroidal anti-inflammatory drugs (NSAIDs), and immunosuppressive drugs [4]. However, recent case reports have implicated DOACs, particularly apixaban, as possible triggers of cutaneous small-vessel vasculitis. Reported cases typically describe the onset of palpable purpura within one to six weeks of starting apixaban, with biopsy-confirmed LCV and resolution following drug discontinuation [3,5-8]. In our case, the temporal association between apixaban initiation and symptom onset, along with improvement of symptoms with the discontinuation of the drug, supports apixaban as the likely etiologic agent.

The pathophysiology of LCV involves the deposition of immune complexes in the walls of small vessels, triggering complement activation and recruitment of neutrophils. This inflammatory response leads to vessel wall damage and fibrinoid necrosis [9]. Notably, consistent findings across several case reports, including those by Nasir et al. [6] and El-Sabbagh et al. [8], have documented negative serological findings such as negative ANCA titers and normal complement levels which was also observed in our case. These consistent findings across cases support the concept that LCV is more often a localized, immune-mediated process rather than a manifestation of systemic vasculitis.

Diagnosis of LCV requires a combination of clinical and histologic evaluation. Cutaneous findings such as palpable purpura, particularly on the lower extremities, with no sign of systemic disease should prompt the consideration of vasculitis, especially in patients with recent medication changes. Further evaluation to rule out infectious, autoimmune, and inflammatory conditions should be done to strengthen the diagnosis of drug-induced LCV [5]. Biopsy of early lesions, within the first 24 hours, typically shows neutrophilic infiltration and fibrinoid necrosis, while in lesions after 48 hours, degeneration of neutrophils leads to a predominance of lymphocytes [4]. For our patient, the biopsy demonstrated only nonspecific perivascular inflammation, likely influenced by prior corticosteroid therapy. Although histology is an important component, the clinical presentation and exclusion of alternative causes supported a presumptive diagnosis of apixaban-induced LCV.

Management of drug-induced LCV depends on disease severity. In mild cases, discontinuing the triggering medication along with supportive care such as leg elevation, compression stockings, and antihistamines may be sufficient. More severe or widespread involvement may warrant a tapering course of corticosteroids over four to six weeks [9]. In our case, corticosteroids were initiated prior to discontinuing apixaban, making it difficult to determine whether symptom resolution was due to steroid treatment, apixaban withdrawal, or both. However, the clinical course and lack of recurrence after transition to rivaroxaban reinforce apixaban as the most likely cause.

## Conclusions

This case highlights the importance of considering drug-induced causes in new-onset vasculitis, particularly in patients recently started on anticoagulants. Although overlapping treatments limit definitive interpretation, the overall clinical course, including temporal association, exclusion of alternative causes, and absence of recurrence on rivaroxaban, is most consistent with apixaban-induced LCV. While rare, this adverse reaction is clinically significant because of its potential for misdiagnosis and unnecessary immunosuppression. Clinicians should maintain a high index of suspicion for drug-induced causes of vasculitis, especially in patients recently initiated on anticoagulation therapy.
